# FMRP binds Per1 mRNA and downregulates its protein expression in mice

**DOI:** 10.1186/s13041-023-01023-z

**Published:** 2023-04-05

**Authors:** Xiangrong Tang, Jing Zhang, Xin Li, Ying Hu, Dengfeng Liu, Jia-Da Li, Renbin Lu

**Affiliations:** 1 Hunan Key Laboratory of Molecular Precision Medicine, Xiangya Hospital, Central South University, Changsha, China; 2 Center for Medical Genetics, School of Life Sciences, Central South University, Changsha, 410078 Hunan China; 3 Center for Reproductive Medicine, Women and Children’s Hospital of Chongqing Medical University, Chongqing, 400010 China; 4 National Clinical Research Center for Geratric Disorder, Xiangya Hospital, Central South University, Changsha, 410008 China; 5 Department of Basic Medical Sciences, Changsha Medical University, Changsha, China

**Keywords:** FXS, FMRP, Circadian rhythm, Per1

## Abstract

FMRP, an RNA-binding protein, has previously shown to be involved in regulation of circadian rhythms in flies and mice. However, the molecular mechanism remains elusive. Here we demonstrate that core circadian component Per1 mRNA was a target of FMRP and the association leads to reduced PER1 expression. In Fmr1 KO mice, the oscillation of PER1 protein expression was significantly affected in a temporal and tissue-dependent pattern when compared to WT mice. Our work thus identified Per1 mRNA as a novel target of FMRP and suggested a potential role of FMRP in regulation of circadian function.

**Supplementary Information:**

The online version contains supplementary material available at 10.1186/s13041-023-01023-z.

## Introduction

Fragile X syndrome (FXS) is the most common form of heritable intellectual disability and the best-known monogenic cause of autism [1]. It is induced by disruption of the fragile X mental retardation 1 gene (FMR1) on the X chromosome and the subsequent absence of FMR protein (FMRP) [2]. Patients with FXS suffer from a range of cognitive and behavioral deficits included social deficits, anxiety, stereotypic movements, hyperactivity, seizures, memory deficits, and sleep dysfunction [3].

FMRP is a multifunctional RNA-binding protein that regulates the translation, transport and stability of downstream target mRNAs essential for regulation of neuronal development and function [4]. Models of FXS in flies and mice exhibit circadian abnormalities in the behavioral rhythm. Mutant flies with *dfmr1* deletion are arrhythmic with respect to the time of eclosion during the day [5]. In addition, a lack of dfmr1 in adult flies also causes arrhythmic locomotor activity, and overexpression of dFmr1 leads to long period of circadian rhythms [6]. Mice lacking FMRP protein display a shorter free-running period of locomotor activity in total darkness [7]. FXR2P share above 60% amino acid identity with FMRP. Interestingly, mice with the absence of both FMRP and FXR2P protein completely abolish the rhythmicity of locomotor activity in a light–dark cycle [7]. Recently, a study reported that a specific loss of FMRP in CA1 pyramidal neurons of the mouse hippocampus results in circadian-dependent defects in learning and memory [8]. These studies strongly suggest that FMRP is necessary to maintain the behavioral circadian rhythms and this role may be involved in the behavioral alterations observed in FXS patients.

Circadian rhythm of daily variations in many physiologic and behavioral variables, including alertness, blood pressure and sleep–wake are driven by endogenous circadian clocks [9–13]. The circadian clocks are composed of interconnected transcription-translation-based negative feedback loop. In mammals, the basic components of circadian locomotor output cycles kaput (CLOCK) forms heterodimers with aryl hydrocarbon receptor nuclear translocator-like protein 1 (ARNTL; also known as BMAL1) to activate transcription of Period (Per1, Per2, Per3) and Cryptochrome (Cry1, Cry2) genes via direct binding to the E-box elements at their promoter regions. PER and CRY proteins heterodimerize and translocate into the nucleus to interact with CLOCK and BMAL1, thus inhibiting their transcriptional activity [14, 15]. The rhythmic activation and repression of E-box-driven transcription generate the endogenous ~ 24-h oscillation of circadian rhythms in mammals [16, 17].

In this study, we sought to understand the molecular pathogenesis of defects in circadian rhythm in FXS and identify potential component of the circadian pathway affected by FMRP. We confirmed that FMRP could bind *Per1* mRNA and suppress its expression. In mice, rhythmic expression of PER1 protein in cortex, hypothalamus and liver of Fmr1 KO mice was significantly affected compared to WT mice and which was in a temporal and tissue-dependent pattern. However, *Fmr1* KO mice show no distinct phenotypes in circadian rhythm of locomotor activity. Our data identify Per1 mRNA as a novel target of FMRP and indicate a potential role of FMRP in regulation of circadian function.

## Materials and methods

### Cell culture and transfection

U2OS were cultured in Dulbecco’s modified Eagle’s medium (DMEM) (Sigma-Aldrich, St. Louis, USA, #D5546) supplemented with 10% fetal bovine serum (FBS) (ThermoFisher Scientific, Massachusetts, USA, #10099), 100 units/ml penicillin, and 100 μg/ml streptomycin at 37 °C in 5% CO_2_ incubators. Plasmid and siRNA transfections were performed with Lipofectamine 2000 (Invitrogen) reagents according to the manufacturer’s protocol.

### Animals

FXS mice were generated by using CRISPR-Cas9 technology. Cas9 mRNA and two guide RNAs (gRNA) targeting the upstream and downstream regions of the mouse Fmr1 gene were injected into C57BL/6 mouse oocytes, and a mouse with deletion of the 2–5 exon was used as a founder. Before behavioral tests, mice of the same sex were group-housed with 3–5 animals per cage under controlled conditions [temperature, 20 ± 2 °C; relative humidity, 50–60%; 12:12-h light–dark (LD) cycle, lights on at 7:00 AM and lights off at 7:00 PM] and had free access to food and water. The genotype was confirmed by PCR. The primers for genotyping were as following:

Fmr1-WT forward primer (F1): 5′-AGTAGTTTGGTTACAGTAGTGAAGG-3′;

Fmr1—Mutant forward primer (F2): 5′-TCACCAAGGTGTGCTACCAATGC-3′;

Fmr1—reverse primer (R): 5′- CTCTAAAAGGGAAAGCATCAGGAG-3′.

All procedures regarding the care and use of animals were approved by the ethics committee of Center for Medical Genetics, School of Life Sciences, Central South University of China. All methods were performed in accordance with approved guidelines.

### RNA co-immunoprecipitation

U2OS Cells were lysed with lysis buffer (20 mmol/L Tris–HCl pH 7.4, 150 mmol/L NaCl, 5 mmol/L MgCl2, 1 mmol/L DTT, 1% Triton X-100) supplemented with RNase inhibitor (Takara, Kusatsu, Japan, #2313A) and proteinase inhibitor cocktail (Sigma-Aldrich, St. Louis, USA, #P8340). Cleared lysates with 1 mg total protein were incubated with Dynabeads Protein G (Invitrogen, Paisley, UK, #10003D) coated by either anti-FMRP antibody (Abcam, Cambridge, UK, #ab259335) for U2OS cells or normal mouse IgGs ( Sigma-Aldrich, Saint Louis, MO, USA, #I5381) overnight at 4 °C, and 10% of the lysates were saved as input. About 30% of the beads were used for Western blot analysis and the rest for mRNA enrichment analysis. RNA was extracted by Trizol (Invitrogen, CA, USA, #15596-026) and reverse-transcribed using the Revert Aid First Strand cDNA Synthesis Kit. Quantitative real-time PCR (qRT-PCR) was performed and the mRNA enrichment was calculated with 18S rRNA as an external control and input for normalization. The primers used were as follows:human-Per1-F: 5′-TGAAGCAAGACCGGGAGAG-3′;human-Per1-R: 5′-CACACACGCCGTCACATCA-3′;

### Circadian behavior analysis

Mice aged 4–6 months were individually housed within cages equipped with running wheels and were allowed free access to food and water. Their locomotor activities were recorded as revolutions per 5-min interval. Mice were entrained to an initial LD cycle (light intensity ∼ 150 lx, lights on at 7:00 AM and lights off at 7:00 PM). After 2–3 weeks of activity recording in 12:12-h light–dark conditions, the mice were placed in constant darkness (DD) for ∼ 3 weeks. These mice were then subjected to a light-induced phase shift at day ~ 20 of DD. Animals in their home cages were moved to another room and exposed to a 15-min pulse of white light (∼ 150 lx) at circadian time (CT) 16, at which CT12 was designated as activity onset. The light induced phase-shift amplitude was derived from regression lines drawn through the activity onset at least 7 days immediately before the day of stimulation and 7 days after reestablishment of a steady-state circadian period after stimulation. The free-run period was calculated using ClockLab software (Actimetrics, Evanston, IL, USA) in the Matlab environment. The free-run period was measured by a 2 periodogram from days 10 through 25 under DD.

### Western blotting

Mice at the age of 4–8 weeks without any behavioral test were sacrificed by cervical dislocation. The cortex, hypothalamus and liver tissues were dissected at 7am and 7 pm. Cells or tissue samples were lysed in SDS lysis buffer (2% SDS, 63 mM Tris–HCl, and 10% glycerol) and the protein concentration was determined using the PierceTM BCA protein Assay kit (Termo Fisher, Waltham mass, USA). Proteins in lysates were separated by SDS-PAGE, transferred to nitrocellulose membranes (PVDF), and immunoblotted with the corresponding antibodies overnight at 4 °C after blocked in 5% skim milk/Tris-buffered saline that contained 0.1% Tween 20 at room temperature for 1 h. Membranes were then washed and incubated with horseradish peroxidase conjugate secondary antibodies. The proteins were visualized using the Pierce™ ECL Western Blotting Substrate kit (Thermo Scientific; 32106). Band intensities were quantified by ImageJ. The antibodies were listed as following: anti-Per1 antibody-N-terminal (1:500, Abcam, Cambridge, UK, #ab136451); anti-FMRP-antibody (1:500, Abcam, Cambridge, UK, #ab259335); anti-β-actin antibody (1:1000, Sigma, USA, A2228).

### Statistical analysis

Statistical analyses were performed using GraphPad Prism 7 (RRID: RDG_1346427 GraphPad Software, lnc., San Diego, CA, USA). All experiments were repeated at least three times and the distribution of data points is presented as mean ± SEM. Student’s t-test for comparison of two conditions or ANOVAs were utilized with post hoc Bonferroni multiple comparisons test for three or more conditions. P < 0.05 was considered significant. All data are presented as the mean ± SEM; *P < 0.05, **P < 0.01, ***P < 0.001, ****P < 0.0001.

## Results

### Identification of FMRP targets

**Fig. 1:**
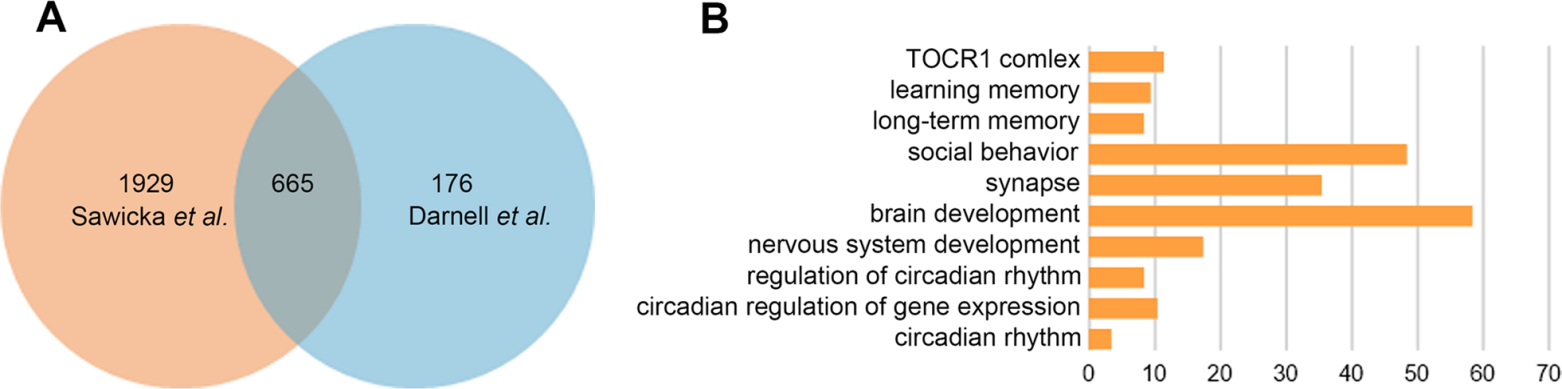
Identification of FMRP targets. **A** Venn diagram of potential FMRP targets identified in two previous study conducted by Sawicka et al. and Darnell et al. by using CLIP technology. **B** Gene ontology in 665 transcripts appeared in both studies

Given the critical role of FMRP in brain function, numerous studies have focused on the transcripts it binds and regulates [18, 19]. Cross-linking immunoprecipitation (CLIP) is a highly efficient, commonly used method to analyze protein interactions with RNA [20]. In present study, we reanalyzed the data from two previous studies on identification of potential FMRP target mRNA in mouse brain by using CLIP technology [8, 21]. As a result, we identified totally 665 transcripts appeared in both studies (Fig. 1A). GO analysis shows that these transcripts are mainly enriched in terms of synapse, brain development, social behavior and nervous system development which consistent with the recognized FMRP function (Fig. 1B). Interestingly, 20 transcripts are enriched in 3 terms associated with circadian rhythm including regulation of circadian rhythm, circadian regulation of gene expression, and circadian rhythm (Table 1). When further analyzed the function of these 20 potential target transcripts and excluded the reported FMRP target transcripts, we finally selected Per1 as a candidate FMRP target transcript.

**Table 1:** Potential targets transcripts of FMRP clustered in circadian rhythm

GO-term	Gene count	Gene
Circadian rhythm	11	SETX, PER1, NTRK2,GSK3B, NCOR1, KCNH7, NRIP1, EP300, ADCY1, PPARGC1A, NPAS2
Circadian regulation of gene expression	9	PER1, NCOA2, EGR1, MAGED1, MYCBP2, NRIP1, HUWE1, PPARGC1A, NPAS2
Regulation of circadian rhythm	8	PRKCG, PER1, GSK3B, MAGED1, USP9X, ADXY1, PPARGC1A, MTOR

### FMRP interacts with core circadian transcript Per1 mRNA

**Fig. 2:**
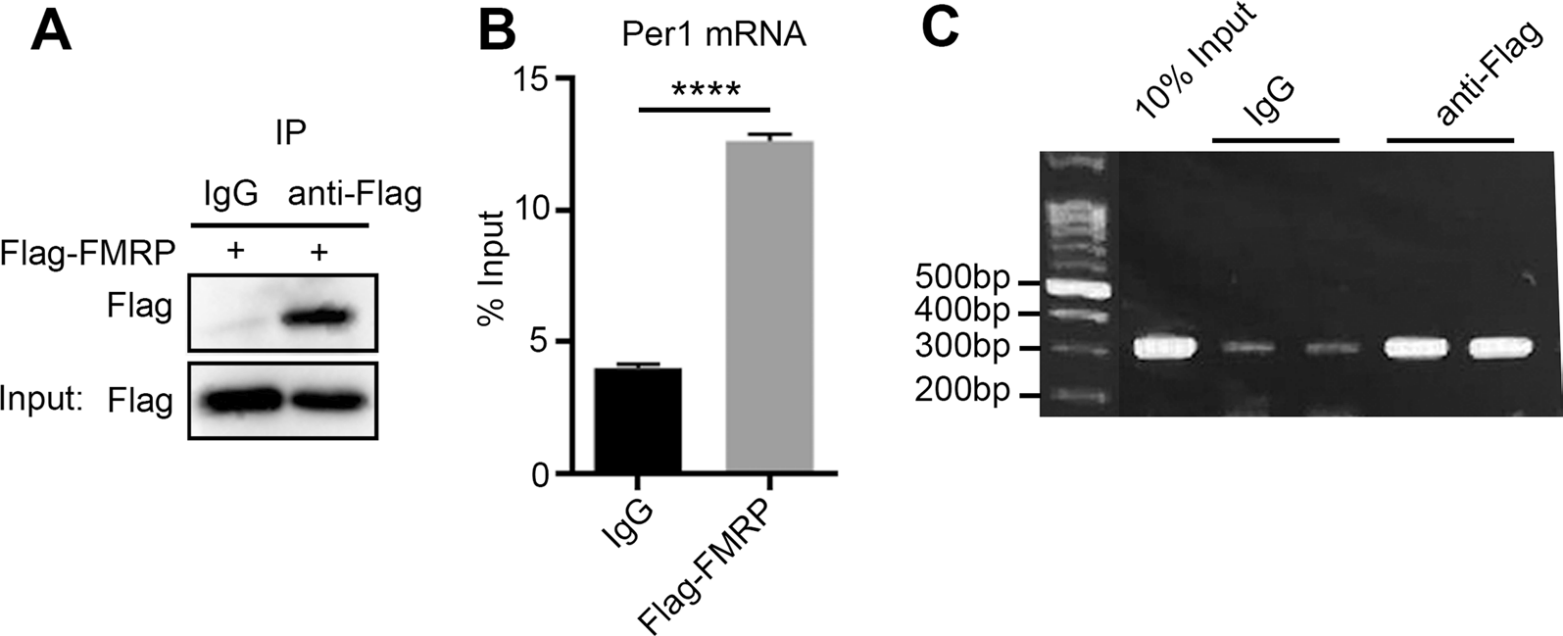
FMRP interacts with core circadian transcript Per1 mRNA. **A** U2OS cells expressing Flag-FMRP were subjected to RIP assay. Immunoblot confirmed the pull-downed Flag-tagged FMRP. **B** RT–qPCR analysis shows binding of FMRP to Per1 mRNA in U2OS cells. Data presented as means ± SEM (n = 3). ****P < 0.0001; two-tailed Student’s t-test. **C** Agarose gel electrophoresis of RT-PCR reactions from RIP assay

Per1 is a core component in the mammalian circadian clockwork and is important to maintenance of circadian rhythms in cells and tissues [22]. To address whether FMRP directly regulates Per1 mRNA, we first performed RNA immunoprecipitation (RIP) assay with an antibody against Flag or control IgG in U2OS cells overexpressed Flag-tagged FMRP. Figure 2A confirms that the anti-Flag antibodies could specifically immunoprecipitate Flag-tagged FMRP. The quantitative PCR and RT-PCR results revealed a high enrichment of Per1 mRNA in the immunocomplex pulled-down by Flag antibody, but not by control IgG (Fig. 2B, C). These data indicated that Per1 mRNA is a potential novel target of FMRP protein in cells.

### FMRP suppress PER1 protein expression in cells

**Fig. 3:**
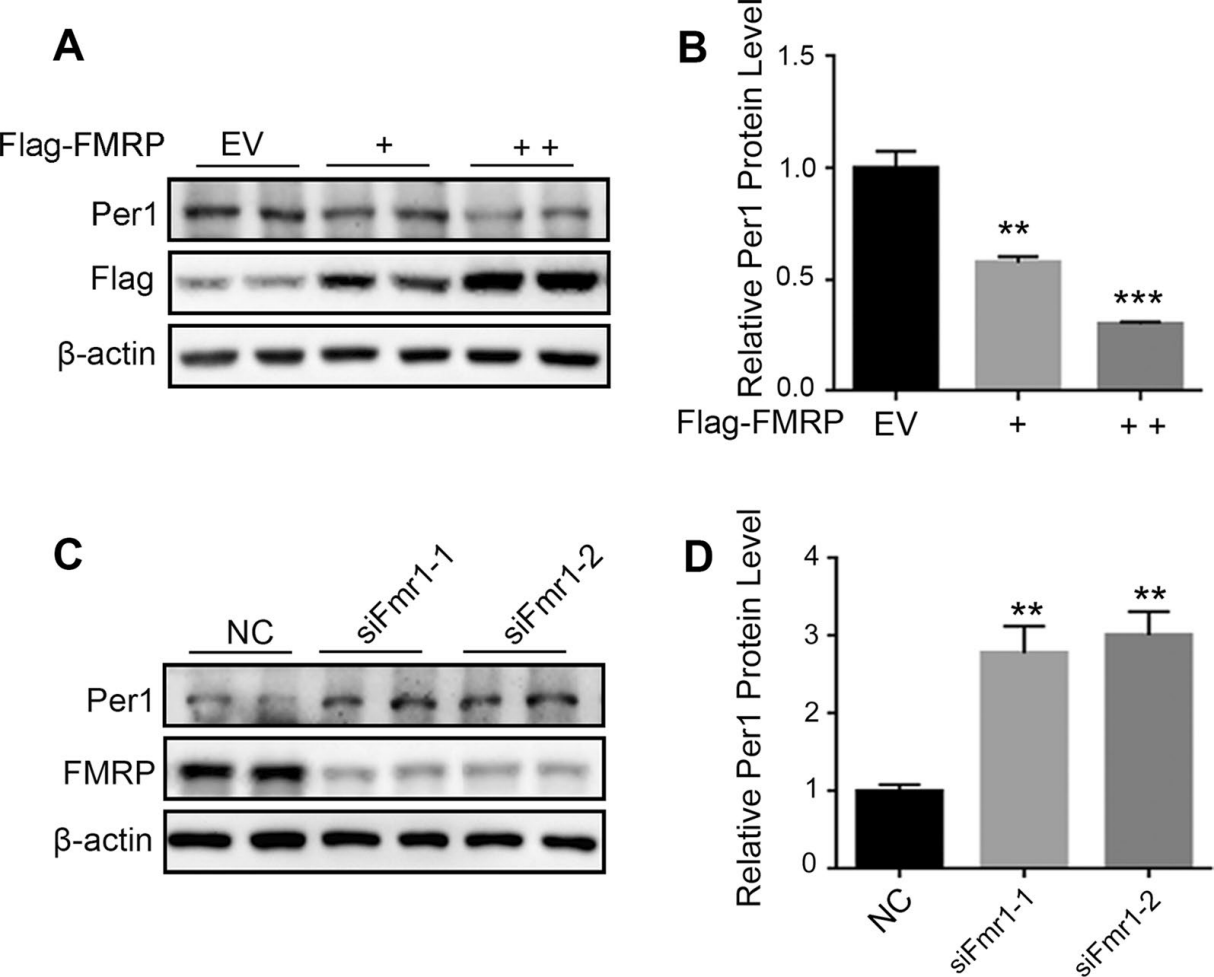
FMRP suppress PER1 protein expression in cells. **A, B** Representative immunoblots (**A**) and statistics data of three independent experiments (**B**) from U2OS cells transfected with control siRNA (NC) or Fmr1 siRNAs. Data are presented as means ± SEM, **P < 0.01, ***P < 0.001 post hoc Dunnett’s t-test, one-way ANOVA. **C, D** Representative immunoblots (**C**) and statistics data of three independent experiments (**D**) from U2OS cells transfected with different doses of Fmr1 or empty vector (EV). Data are presented as means ± SEM, **P < 0.01, post hoc Dunnett’s t-test, one-way ANOVA

FMRP can bind its target mRNA and generally act as a translational repressor. Thus far, our experiments indicate that FMRP can interact with the Per1 mRNA, but the functional role of this interaction is not clarified. To assess the consequence of this interaction on Per1 mRNA level, we knockdown FMRP by transient transfection of siRNAs. Silencing *Fmr1* gene significantly increased the Per1 mRNA level as analyzed by qPCR (Additional file 1: Figure S1). We also detect the effect of FMRP on Per1 protein expression. Consistantly, silencing *Fmr1* gene significantly increased Per1 protein expression (Fig. 3A, B) and conversely, overexpression of *Fmr1* decreased Per1 protein expression (Fig. 3C, D). These results suggest FMRP suppress PER1 protein expression in cells.

### FMRP regulates PER1 protein expression in a temporal and tissue-dependent patterns in mice

**Fig. 4:**
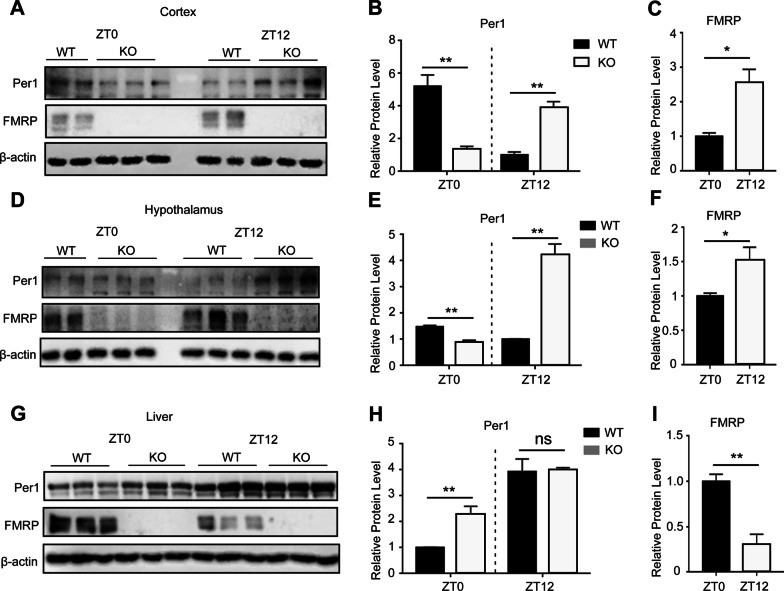
FMRP regulates PER1 protein expression in a temporal and tissue-dependent patterns in mice. **A–C** Western blot analysis of cortex lysates from WT and Fmr1 KO mice taken at the ZT0 and ZT12 of a day (**A**) and the quantification of Per1 (**B**) and FMRP (**C**) protein levels; Data are presented as means ± SEM; *P < 0.05; **P < 0.01; two-tailed Student’s t-test; n = 4 mice/genotype/time point. **D–F** Western blot analysis of hypothalamus lysates from WT and Fmr1 KO mice taken at the ZT0 and ZT12 of a day (**A**) and the quantification of Per1 (**B**) and FMRP (**C**) protein levels; Data are presented as means ± SEM; *P < 0.05; **P < 0.01; two-tailed Student’s t-test; n = 4 mice/genotype/time point. **G–I** Western blot analysis of liver lysates from WT and Fmr1 KO mice taken at the ZT0 and ZT12 of a day (**A**) and the quantification of Per1 (**B**) and FMRP (**C**) protein levels; Data are presented as means ± SEM; ns: P > 0.05; **P < 0.01; two-tailed Student’s t-test; n = 4 mice/genotype/time point

To elucidate the physiological function of FMR1 in vivo, we generated a mouse strain with a deletion of *Fmr1* gene (Additional file 1: Figure S2). In cortex and hypothalamus, PER1 protein expression in Fmr1 KO mice was significantly reduced compared to WT mice at ZT0 (Zeitgeber 0) when FMRP protein was at low-expression level, while PER1 protein expression was increased at ZT12 when FMRP protein was at high-expression level (Fig. 4A–F). However, in peripheral liver tissue, PER1 protein expression was significantly increased compared to WT mice at ZT0 when FMRP protein was at high-expression level in the WT mice, but no significantly change was observed between WT and Fmr1 KO mice at ZT12 when FMRP protein was at low-expression level in the WT mice (Fig. 4G–I). The above findings indicate that FMRP deficiency leads to significant changes in rhythmic expressions of PER1 protein and this consequence shows temporal and tissue-specific effects.

To address the function of FMRP in circadian regulation, we monitored the wheel-running activity of Fmr1 KO mice and their WT littermate controls. Both WT and Fmr1 KO mice entrained to LD cycles and showed no significant differences in daily counts or amplitudes of locomotor rhythmicity (Additional file 1: Figure S3A). Under DD, both WT and Fmr1 KO mice showed similar free-running periods (Additional file 1: Figure S3A-B). We also compared the phase shifts generated by exposure to a brief light pulse at CT16 (white light, ∼ 150 lx, 10 min) under DD conditions. In response to this treatment, we did not see any significant difference between WT and Fmr1 KO mice (Additional file 1: Figures S3C-D). Our data thus demonstrate that *Fmr1* KO mice have normal phenotypes in circadian rhythm of locomotor activity.

## Discussion

FXS as the most common cause of inherited intellectual disability, results from the loss of the FMRP protein expression [2, 23]. Given the critical role of FMRP in neuronal development, its physiological target transcripts were extensively studied. Previous studies suggested that Per1 mRNA might bind to FMRP using CLIP technology in mouse brain tissues [8, 21]. In our study, we provided molecular evidence for that FMRP specifically interacts with Per1 mRNA and disruption of this interaction results in aberrant Per1 mRNA level and Per1 mRNA translation. Thus, our results identified Per1 mRNA as a new target for FMRP.

The main function of FMRP is to interact with target mRNA and commonly act as a repressor of target mRNA translation [4, 24]. Our data indicated that deletion of Fmr1 expression led to aberrant Per1 protein expression in cells and tissues, suggesting FMRP affected the translation of Per1 mRNA. In fact, FMRP regulates mRNA translation in various ways. FMRP can reversibly stall ribosomes specifically on its target mRNAs in the process of translation. In FMRP loss-function mouse model, ribosomal stalling on FMRP target transcripts is relieved and protein expression is significantly increased in the brain [18]. FMRP can regulate the binding of mRNA to ribosome by binding to target mRNA through the G-quartets that is ubiquitous on mRNA [25]. Cells derived from FXS patients display abnormal polyribosome profiles, which indicates that the absence of FMRP alters translation [26]. FMRP can also directly bind to ribosomes to inhibit mRNA translation. In details, FMRP binds within the intersubunit space of the 80 s ribosome which would results in a blockage of the binding of tRNA and translation elongation factors on the ribosome, thereby reducing protein translation [27]. Therefore, although our data indicated that FMRP binds to Per1 mRNA to regulate its translation, how FMRP functions in this process requires further investigation.

There is increasing evidence that the absence of FMRP leads to tissue and cell-type specific deficits. For example, several studies reported that extracellular signal-regulated kinase (ERK) and mechanistic target of rapamycin (mTOR) signaling was disrupted in the FMRP deficient mice while the effect on these pathways was different between hippocampus and the cortex [28]. ERK was aberrant deactivated following mGluR stimulation in cortex of Fmr1 KO mice, whereas it was illustrated to be normal in hippocampal tissue [29]. mTOR activity was found normal at synapses of the neocortex of Fmr1 KO mice, whereas it is elevated at synapses of the hippocampus. Interestingly, our data shows that PER1 protein expression was significantly reduced at ZT0 in cortex and hypothalamus, while it is significantly increased in peripheral liver tissue of Fmr1 KO mice. These results suggest that FMRP may regulate the expression of Per1 in a tissue-dependent patterns in mice. In addition, our data also indicates that though PER1 protein expression was significantly increased compared to WT mice at ZT0, no significantly change was observed between WT and Fmr1 KO mice at ZT12 when FMRP protein was at low-expression level in the WT mice. Our data support the speculation that FMRP may affect the expression phase of Per1 protein.

Two studies reported by *Dockendorff* et al. and Morales et al. demonstrated that although the rhythmic mRNA and protein expressions of the core clock genes *per* and *tim* was normal in the FXS Drosophila melanogaster, FMRP deficiency results in behavioral phenotypes of FXS Drosophila melanogaster including arrhythmic eclosion and locomotor activity [5, 6]. In mammals, mice lacking FMRP display a slightly shorter free-running period of locomotor activity in total darkness [7]. Additionally, loss of FMRP in CA1 pyramidal neurons of the mouse hippocampus results in circadian-dependent defects in learning and memory [8]. In our study, we have carried out behavioral paradigms to detect the circadian rhythm phenotypes of FXS mice under LD or DD condition, however, no obvious abnormality has been detected. In our study, we conducted rhythmic behavior testing with 4–6 months old mice. In general, at 4–6 months mice, their rhythmic behavior tends to be stable and we think it is suitable for conducting rhythmic behavior testing at this age. Despite all this, there are still a little limitation in our experimental design and the rhythmic behavior of mice aged 4–8 weeks requires further investigation. It also should be noted that the circadian clocks control many of output pathways such as aging, feeding-fasting, glucose metabolism, immune function and sleep-wakefulness [16, 30–32]. We will carry out other behavioral paradigms in future to detect whether circadian phase of specific behavior might be influenced.

Collectively, our findings indicate Per1 mRNA as a new target for FMRP and that FMRP regulates PER1 protein expression in a circadian phase and tissue dependent pattern. The specific phenotypes associated FXS may arise from the disruption of the interaction of FMRP with Per1 mRNA.

## Supplementary Information

**Additional file 1.** Supplemental data information.

## Data Availability

No data was used for the research described in the article.

## References

[1] Hagerman RJ, Berry-Kravis E, Hazlett HC, Bailey DB, Moine H, Kooy RF, Tassone F, Gantois I, Sonenberg N, Mandel JL (2017). Fragile X syndrome. Nat Rev Dis Primers.

[2] Richter JD, Zhao X (2021). The molecular biology of FMRP: new insights into fragile X syndrome. Nat Rev Neurosci.

[3] Deng PY, Klyachko VA (2021). Channelopathies in fragile X syndrome. Nat Rev Neurosci.

[4] Darnell JC, Klann E (2013). The translation of translational control by FMRP: therapeutic targets for FXS. Nat Neurosci.

[5] Inoue S, Shimoda M, Nishinokubi I, Siomi MC, Okamura M, Nakamura A, Kobayashi S, Ishida N, Siomi H (2002). A role for the Drosophila fragile X-related gene in circadian output. Curr Biol.

[6] Dockendorff TC, Su HS, McBride SM, Yang Z, Choi CH, Siwicki KK, Sehgal A, Jongens TA (2002). Drosophila lacking dfmr1 activity show defects in circadian output and fail to maintain courtship interest. Neuron.

[7] Zhang J, Fang Z, Jud C, Vansteensel MJ, Kaasik K, Lee CC, Albrecht U, Tamanini F, Meijer JH, Oostra BA (2008). Fragile X-related proteins regulate mammalian circadian behavioral rhythms. Am J Hum Genet.

[8] Sawicka K, Hale CR, Park CY, Fak JJ, Gresack JE, Van Driesche SJ, Kang JJ, Darnell JC, Darnell RB (2019). FMRP has a cell-type-specific role in CA1 pyramidal neurons to regulate autism-related transcripts and circadian memory. Elife.

[9] Nassan M, Videnovic A (2022). Circadian rhythms in neurodegenerative disorders. Nat Rev Neurol.

[10] Smolensky MH, Hermida RC, Portaluppi F (2017). Circadian mechanisms of 24-hour blood pressure regulation and patterning. Sleep Med Rev.

[11] Huang W, Ramsey KM, Marcheva B, Bass J (2011). Circadian rhythms, sleep, and metabolism. J Clin Invest.

[12] Saper CB, Scammell TE, Lu J (2005). Hypothalamic regulation of sleep and circadian rhythms. Nature.

[13] Challet E (2019). The circadian regulation of food intake. Nat Rev Endocrinol.

[14] Takahashi JS (2017). Transcriptional architecture of the mammalian circadian clock. Nat Rev Genet.

[15] Hastings MH, Maywood ES, Brancaccio M (2018). Generation of circadian rhythms in the suprachiasmatic nucleus. Nat Rev Neurosci.

[16] Patke A, Young MW, Axelrod S (2020). Molecular mechanisms and physiological importance of circadian rhythms. Nat Rev Mol Cell Biol.

[17] Golombek DA, Rosenstein RE (2010). Physiology of circadian entrainment. Physiol Rev.

[18] Darnell JC, Van Driesche SJ, Zhang C, Hung KY, Mele A, Fraser CE, Stone EF, Chen C, Fak JJ, Chi SW (2011). FMRP stalls ribosomal translocation on mRNAs linked to synaptic function and autism. Cell.

[19] Kurosaki T, Mitsutomi S, Hewko A, Akimitsu N, Maquat LE (2022). Integrative omics indicate FMRP sequesters mRNA from translation and deadenylation in human neuronal cells. Mol Cell.

[20] Wang T, Xiao G, Chu Y, Zhang MQ, Corey DR, Xie Y (2015). Design and bioinformatics analysis of genome-wide CLIP experiments. Nucleic Acids Res.

[21] Darnell JC, Fraser CE, Mostovetsky O, Stefani G, Jones TA, Eddy SR, Darnell RB (2005). Kissing complex RNAs mediate interaction between the Fragile-X mental retardation protein KH2 domain and brain polyribosomes. Genes Dev.

[22] Kume K, Zylka MJ, Sriram S, Shearman LP, Weaver DR, Jin X, Maywood ES, Hastings MH, Reppert SM (1999). mCRY1 and mCRY2 are essential components of the negative limb of the circadian clock feedback loop. Cell.

[23] Bagni C, Zukin RS (2019). A synaptic perspective of fragile X syndrome and autism spectrum disorders. Neuron.

[24] Darnell JC, Mostovetsky O, Darnell RB (2005). FMRP RNA targets: identification and validation. Genes Brain Behav.

[25] Kenny PJ, Kim M, Skariah G, Nielsen J, Lannom MC, Ceman S (2020). The FMRP-MOV10 complex: a translational regulatory switch modulated by G-Quadruplexes. Nucleic Acids Res.

[26] Brown V, Jin P, Ceman S, Darnell JC, O'Donnell WT, Tenenbaum SA, Jin X, Feng Y, Wilkinson KD, Keene JD (2001). Microarray identification of FMRP-associated brain mRNAs and altered mRNA translational profiles in fragile X syndrome. Cell.

[27] Chen E, Sharma MR, Shi X, Agrawal RK, Joseph S (2014). Fragile X mental retardation protein regulates translation by binding directly to the ribosome. Mol Cell.

[28] Gantois I, Khoutorsky A, Popic J, Aguilar-Valles A, Freemantle E, Cao R, Sharma V, Pooters T, Nagpal A, Skalecka A (2017). Metformin ameliorates core deficits in a mouse model of fragile X syndrome. Nat Med.

[29] Kim SH, Markham JA, Weiler IJ, Greenough WT (2008). Aberrant early-phase ERK inactivation impedes neuronal function in fragile X syndrome. Proc Natl Acad Sci U S A.

[30] Manoogian ENC, Panda S (2017). Circadian rhythms, time-restricted feeding, and healthy aging. Ageing Res Rev.

[31] Acosta-Rodriguez VA, Rijo-Ferreira F, Green CB, Takahashi JS (2021). Importance of circadian timing for aging and longevity. Nat Commun.

[32] Poggiogalle E, Jamshed H, Peterson CM (2018). Circadian regulation of glucose, lipid, and energy metabolism in humans. Metabolism.

