# Supplementary material for: FMRP binds Per1 mRNA and downregulates its protein expression in mice
Source: Mol Brain. 2023 Apr 5;16:33. doi: 10.1186/s13041-023-01023-z (PMC10077598; doi:10.1186/s13041-023-01023-z)
Supplement: Supplementary file 1 — Additional file 1. Supplemental data information. [file 13041_2023_1023_MOESM1_ESM.docx]

**Supplemental data information**


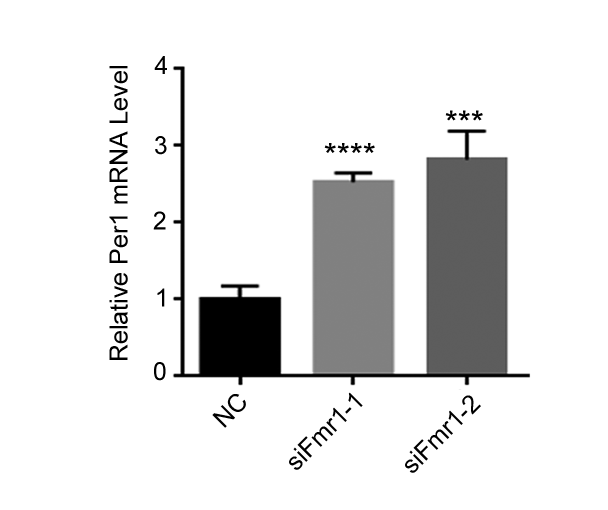


**Figure S1** FMRP regulates expression of core circadian transcript Per1 mRNA **(A)** Disruption of Fmr1 expression in U2OS cells increased the mRNA level of Per1 as assayed by quantitative PCR. Data are presented as means ± SEM; ***P < 0.001; ****P < 0.0001; two-tailed Student's t-test.

**
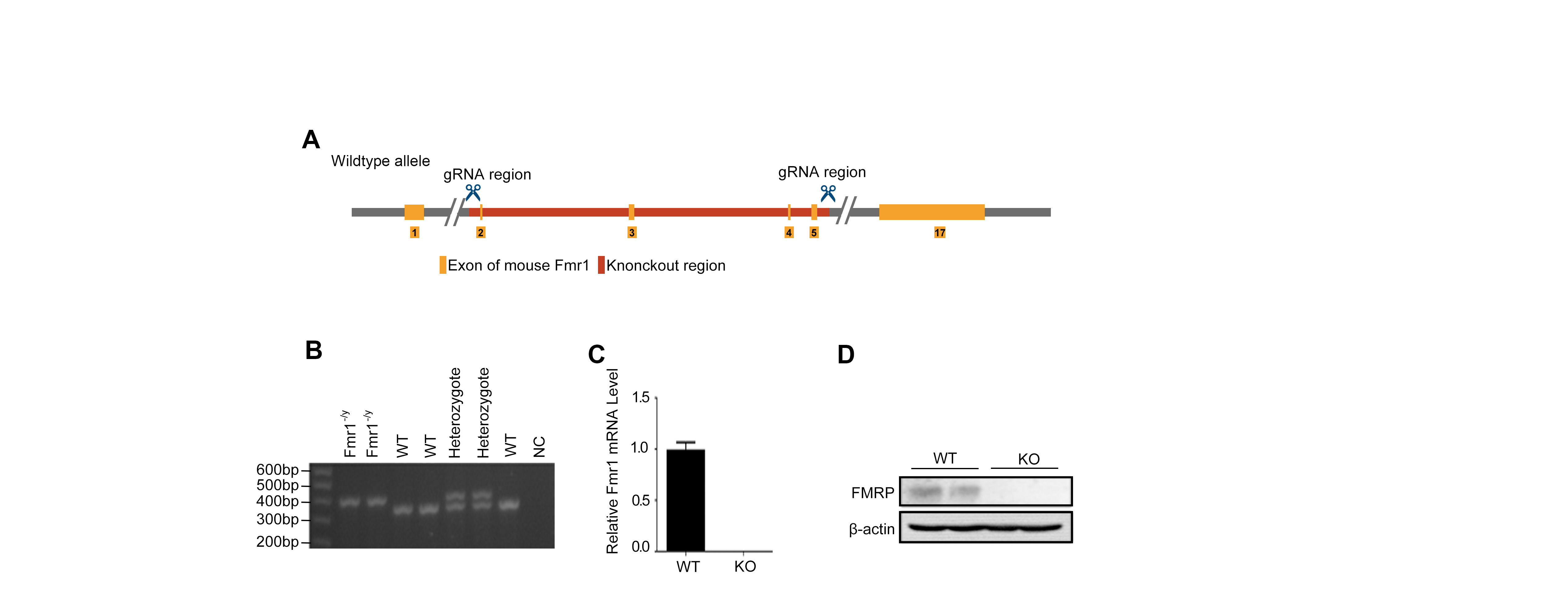
**

**Figure S2** The generation of Fmr1 knock out mouse model. **(A)** CRISPR-cas9 technology was used to generate Fmr1 KO mice. Two gRNAs targeting the Fmr1 gene were injected with Cas9 mRNA into mouse oocytes, resulting in deletion of the region between exon 2 and exon 5 in Fmr1 gene. **(B)** Genotyping by PCR. **(C)** Quantitative PCR of Fmr1 mRNA in the cortex of WT and Fmr1 KO mice. **(D)** FMRP expression in the cortex of WT and Fmr1 KO mice as assayed by Western blot.


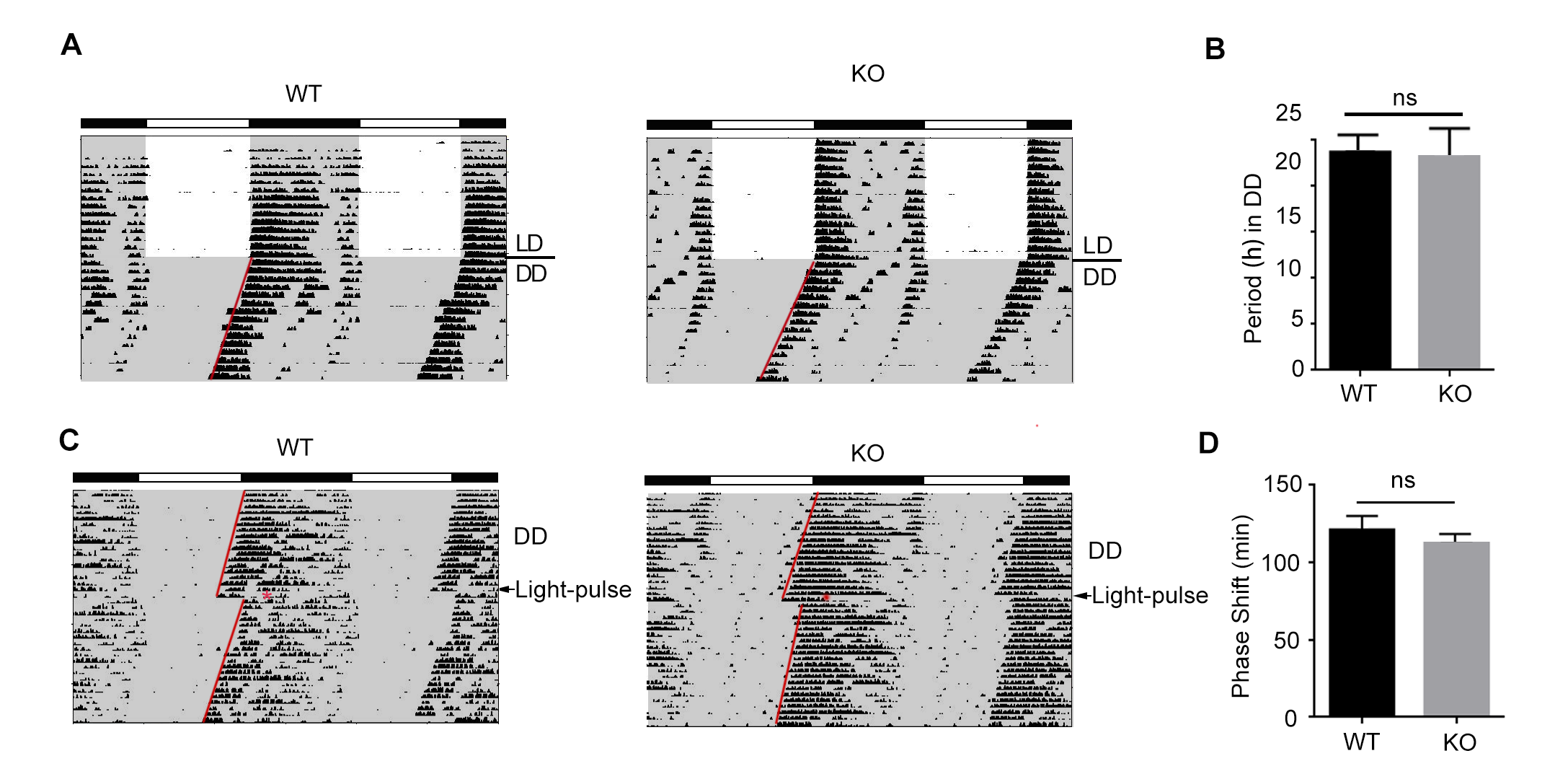


**Figure S3** Fmr1 KO mice shows no phenotypes in circadian behaviors. **(A)** Representative double-plotted actograms of daily wheel-running activity of WT and Fmr1 KO mice. Animals were initially housed in 12:12-h light-dark (LD) conditions and then transferred to constant darkness (DD). The shift from LD to DD is indicated. **(B)** Statistics data of the free-running period of WT and Fmr1 KO mice in DD. Data are presented as means ± SEM.; ns: no significance, p > 0.05, Student’s t-test; WT, n = 6, KO, n=6. **(C)** Representative double-plotted actograms of daily wheel-running activity of WT and Fmr1 KO mice after a light pulse at CT16 during DD. **(D)** Statistics data of the phase shift after a light pulse at CT16 of WT and Fmr1 KO mice in DD. Data are presented as means ± SEM; ns: no significance, p > 0.05, Student’s t-test; WT, n = 6, KO, n=6.
